# Intraocular Inflammation Following Faricimab Intravitreal Injection Treated With Sub-tenon Triamcinolone Acetonide Injection

**DOI:** 10.7759/cureus.83764

**Published:** 2025-05-08

**Authors:** Shinnosuke Yasuda, Hiroki Tsujinaka, Takeyuki Nishiyama, Yutaro Mizusawa, Tetsuo Ueda

**Affiliations:** 1 Ophthalmology, Nara Medical University, Kashihara, JPN

**Keywords:** age-related macular degeneration, diabetic macular edema, faricimab, intraocular inflammation, intravitreal injection, sub-tenon triamcinolone acetonide injection

## Abstract

We report the management of intraocular inflammation (IOI) following faricimab intravitreal injection. Severe IOI was observed in three eyes (one eye with age-related macular degeneration (AMD) and two eyes in the same patient with diabetic macular edema (DME)). These three eyes had fine keratic precipitates, inflammatory cells in the anterior chamber, and vitreous opacity, with decreased best-corrected visual acuity (BCVA) in all patients. Blood tests did not reveal any abnormalities, and anterior chamber multiplex polymerase chain reaction and bacterial cultures were negative. Sub-tenon triamcinolone acetonide (STTA) injections improved anterior inflammation, vitreous opacity, and BCVA, although posterior iris synechia caused by inflammation persisted in one patient. Faricimab injection can induce IOI. In this study, IOI was managed with a STTA injection during the early stage of uveitis. Severe inflammation can lead to complications such as posterior iris synechia.

## Introduction

Diabetic macular edema (DME) and neovascular age-related macular degeneration (nAMD) are leading causes of vision loss globally and are commonly treated with intravitreal injections (injections directly into the eye) of anti-vascular endothelial growth factor (VEGF) agents [1,2]. Anti-VEGF agents work by blocking a protein that promotes abnormal blood vessel growth and leakage in the retina. Faricimab is a newer, bispecific antibody that targets VEGF-A and angiopoietin-2 (Ang-2), another protein involved in blood vessel instability [3]. In Japan, faricimab was approved in 2022 for age-related macular degeneration (AMD) and DME and in 2024 for macular edema secondary to retinal vein occlusion; thus, it is now widely used. While generally effective, there are emerging reports on intraocular inflammation (IOI) as a side effect of faricimab [4-6]. Clinical trials reported IOI incidences per person ranging from 0.3% to 0.9% for nAMD (TENAYA/LUCERNE trials) [7] and 0.3-1.0% for DME (YOSEMITE/RHINE trials) [8], although emerging real-world data suggest the incidence might be higher [9,10]. The characteristics, severity, and treatment of IOI associated with the side effects of faricimab remain unclear. Early recognition and appropriate treatment of IOI are critical to prevent irreversible vision loss. Corticosteroid therapy, such as sub-tenon triamcinolone acetonide (STTA) injections, has shown effectiveness in managing inflammation [4-6]. However, there is no consensus on how and when steroids should be administered.

Moreover, differentiating IOI from infectious etiologies such as herpetic uveitis remains a diagnostic challenge, especially when patients present with keratic precipitates and elevated intraocular pressure (IOP) [11].

Here, we report two cases of IOI after intravitreal injection of faricimab with negative anterior chamber multiplex polymerase chain reaction (PCR) testing for herpesviruses and their management with STTA injections.

## Case presentation

Case 1

**Figure 1:**
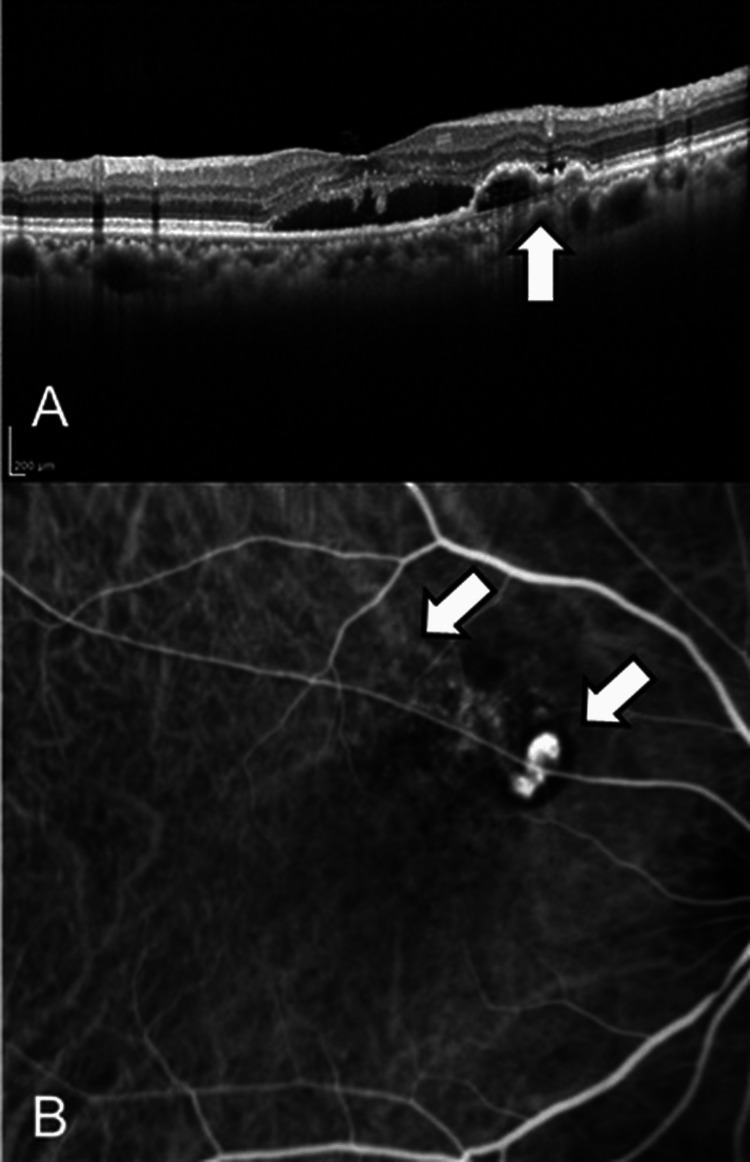
OCT and ICGA images at the initial visit The OCT image (A) shows a double-layer sign (white arrow), suggesting the presence of type I macular neovascularization. ICGA image (B) shows polypoid lesions suggesting dilated vessels of AMD lesion and feeding vessels (white arrows). Central retinal thickness was 266 µm. ICGA: indocyanine green angiography; OCT: optical coherence tomography; AMD: age-related macular degeneration

A 75-year-old woman presented to our hospital with the chief complaint of decreased vision and distorted vision in her right eye. She was diagnosed with AMD in her right eye. The initial best-corrected visual acuity (BCVA) was 20/25, and the IOP was 13 mmHg in the right eye. OCT and indocyanine green angiography revealed polypoidal choroidal vasculopathy in the right eye (Figure 1).

**Figure 2:**
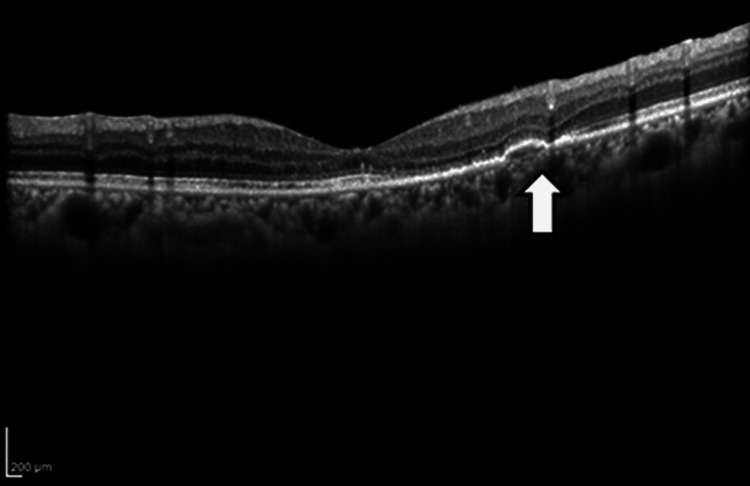
OCT image after three faricimab injections Reduced polyp lesion and subretinal fluid are observed (white arrow). Central retinal thickness was 162 µm. OCT: optical coherence tomography

The patient received three consecutive monthly intravitreal injections of faricimab. After the injections, BCVA was 20/40 in the right eye, and visual improvement was not achieved; however, subretinal fluid reduction and reduced polyp lesions were observed (Figure 2).

**Figure 3:**
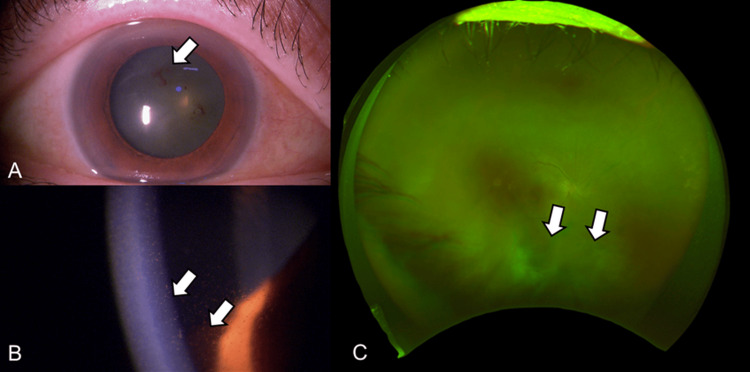
Slit-lamp microscopy (A, B) and fundus (C) images before STTA injection Slit-lamp microscopy shows inflammation of the anterior chamber, including the posterior synechia (A, white arrow), and numerous fine keratic precipitates with pigmentation (B, white arrows). Significant vitreous opacity is also observed (C, white arrows). STTA: sub-tenon triamcinolone acetonide

No complications were observed. After two months, the fourth faricimab intravitreal injection was administered. Two weeks later (17 days post-injection), she noticed decreased vision and floaters in the right eye; BCVA was 20/80 with decreased vision, and the IOP was elevated to 24 mmHg in the right eye. Examination of the anterior segment revealed anterior chamber cells (grade 2+ according to Standardization of Uveitis Nomenclature (SUN) criteria) and numerous fine keratic precipitates, including pigmentation (Figure 3).

**Figure 4:**
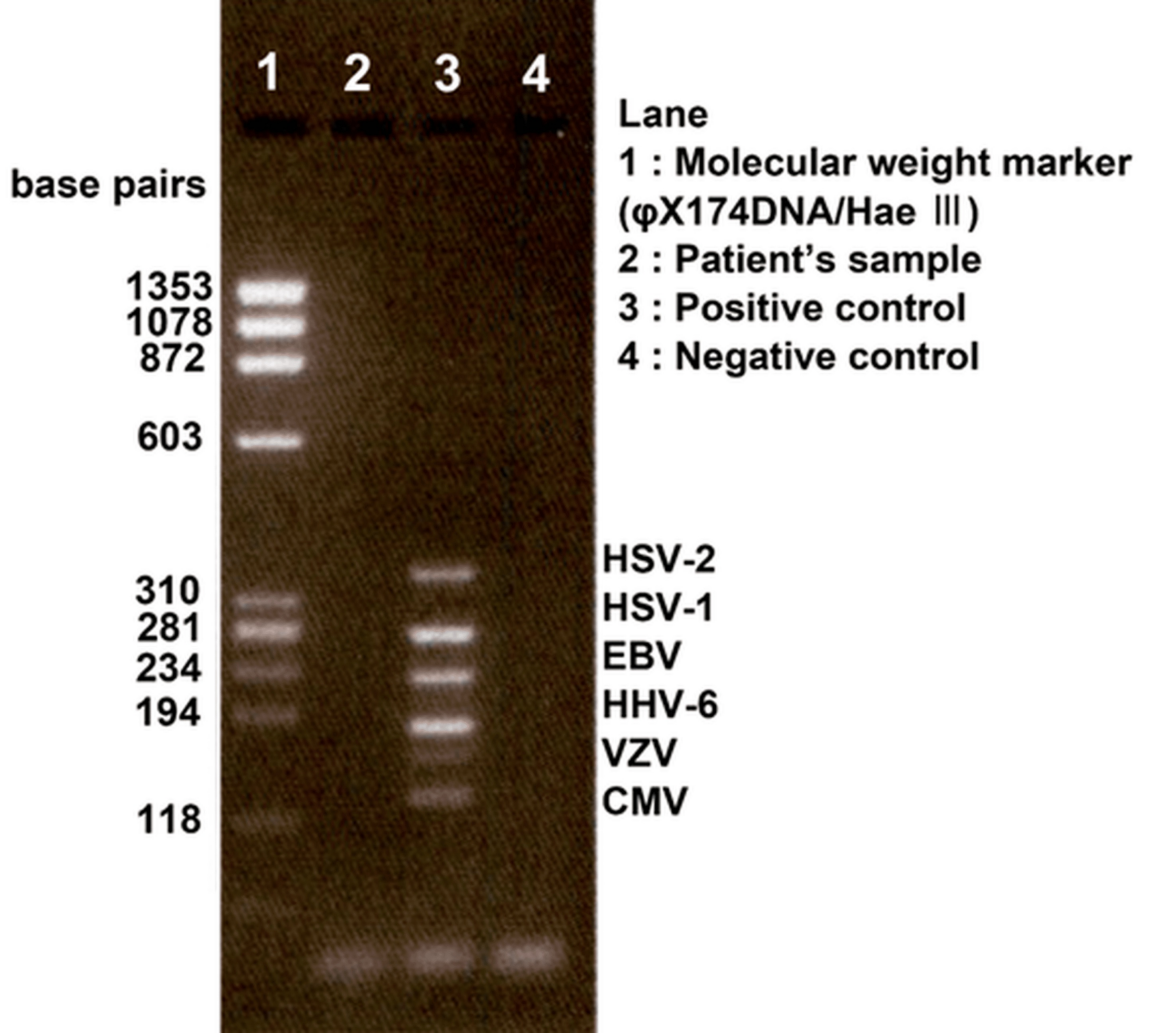
Results of herpes virus multiplex PCR in Case 1 PCR results show that herpes virus is negative in Case 1. PCR: polymerase chain reaction; HSV: herpes simplex virus; EBV: Epstein–Barr virus; HHV: human herpesvirus; VZV: varicella-zoster virus; CMV: cytomegalovirus

Vitreous opacity (grade 3+ according to SUN criteria) was observed, and IOI was diagnosed (Figure 3). The blood tests showed no abnormalities, and the anterior chamber multiplex PCR and bacterial cultures were negative (Figure 4).

**Figure 5:**
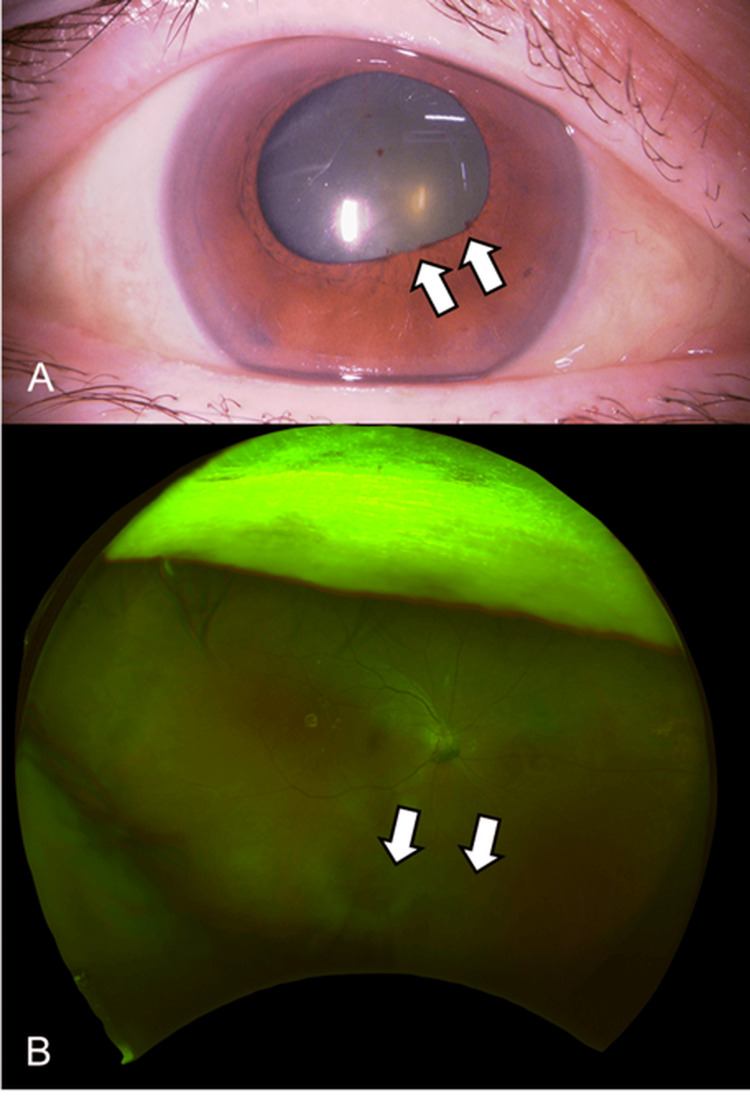
Slit-lamp microscopy (A) and fundus (B) images one month after STTA injection Vitreous opacity is improved (white arrows), although posterior iris synechia is persisting after the resolution of the inflammation (white arrows). STTA: sub-tenon triamcinolone acetonide

An STTA injection of 20 mg was administered to the right eye on the same day, and betamethasone eye drops (0.1%) three times daily were started. Gradually, the anterior segment inflammation and vitreous opacity improved (Figure 5). One month later, BCVA was 20/25 and the IOP was 17 mmHg in the right eye; visual acuity had improved. However, because of intense inflammation, posterior iris synechia remained even after the resolution of the inflammation (Figure 5).

Case 2 (a patient with IOI in both eyes)

**Figure 6:**
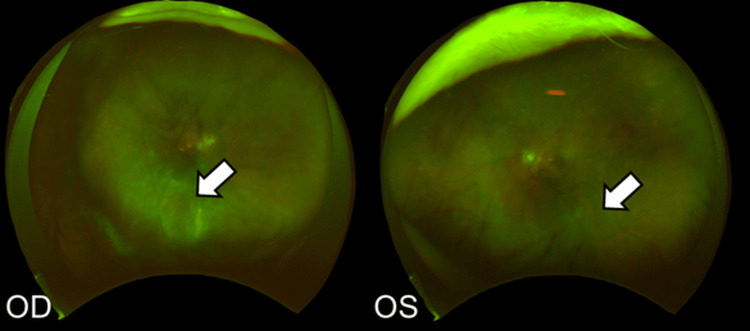
Fundus images two weeks after the third faricimab injection Vitreous opacity is observed in the OD (white arrow) and slightly in the OS (white arrow). OD: oculus dexter; OS: oculus sinister

A 76-year-old man with a history of diabetes mellitus, hypertension, and bronchiectasis presented to our hospital with the chief complaint of decreased vision in both eyes. He was diagnosed with moderate non-proliferative diabetic retinopathy and cataracts in both eyes. The initial BCVA was 20/320 in the right eye and 20/400 in the left eye, and the IOP was 15 mmHg in both eyes. Owing to DME in both eyes, he received three intravitreal ranibizumab injections in each eye. DME was improved by the injections, although it did not resolve completely. Six months later, cataract surgery was performed in both eyes. Two weeks postoperatively, BCVA improved to 20/50 in both eyes. For the persistent macular edema, an intravitreal aflibercept injection was administered to both eyes. One month later, as the macular edema persisted, faricimab intravitreal injections were administered monthly to both eyes for two months. DME improved significantly from the faricimab injections compared to the injection of other anti-VEGF drugs, and a third faricimab injection was administered to both eyes. Two weeks after the injection (14 days post-injection), vitreous opacity was observed in both eyes (the right eye is grade 3+, and the left eye is grade 2+ according to SUN criteria) (Figure 6).

**Figure 7:**
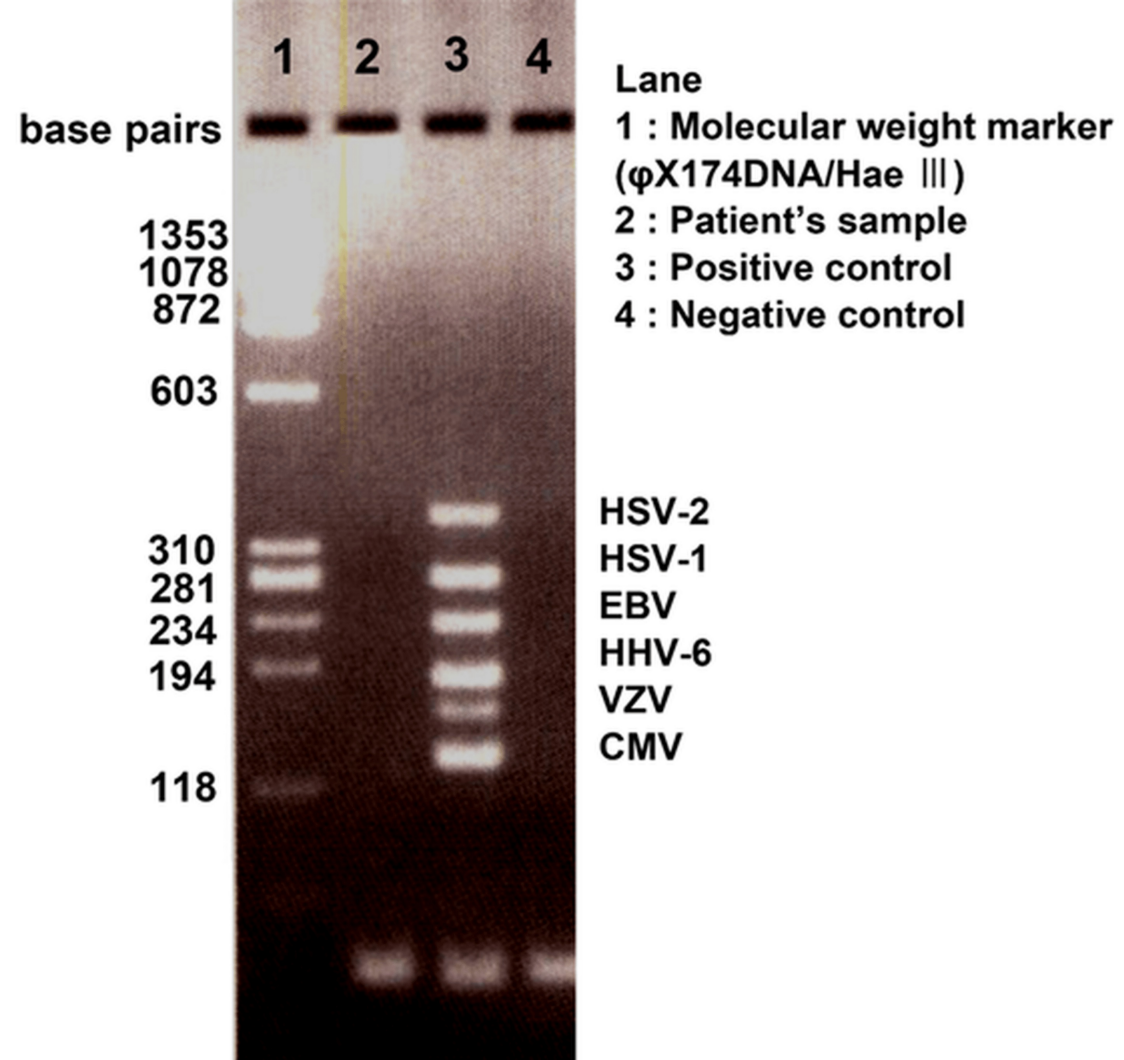
Results of herpes virus multiplex PCR in Case 2 PCR results show that herpes virus is negative in Case 2. PCR: polymerase chain reaction; HSV: herpes simplex virus; EBV: Epstein–Barr virus; HHV: human herpesvirus; VZV: varicella-zoster virus; CMV: cytomegalovirus

BCVA decreased to 20/200 in the right eye and 20/80 in the left eye, and the IOP increased to 27 mmHg in the right eye and 25 mmHg in the left eye. The slit-lamp examination revealed inflammatory findings, including grade 2+ cells (SUN criteria) in the anterior chamber and numerous fine keratic precipitates. The blood test results showed no abnormalities. PCR testing for herpes virus and bacterial culture of the aqueous humor from the right eye were negative (Figure 7).

**Figure 8:**
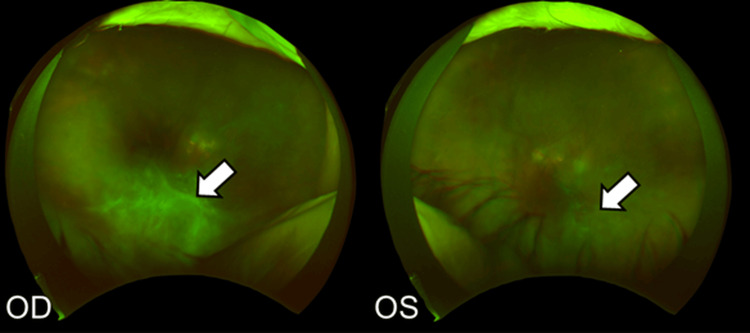
Fundus images four weeks after the third faricimab injection Vitreous opacity is worsening in both eyes (white arrows). OD: oculus dexter; OS: oculus sinister

Betamethasone eye drops (0.1%) three times daily were started in both eyes on the same day. Two weeks later, the anterior segment inflammation slightly improved, although the vitreous opacity was observed in both eyes, predominantly in the right eye (Figure 8).

**Figure 9:**
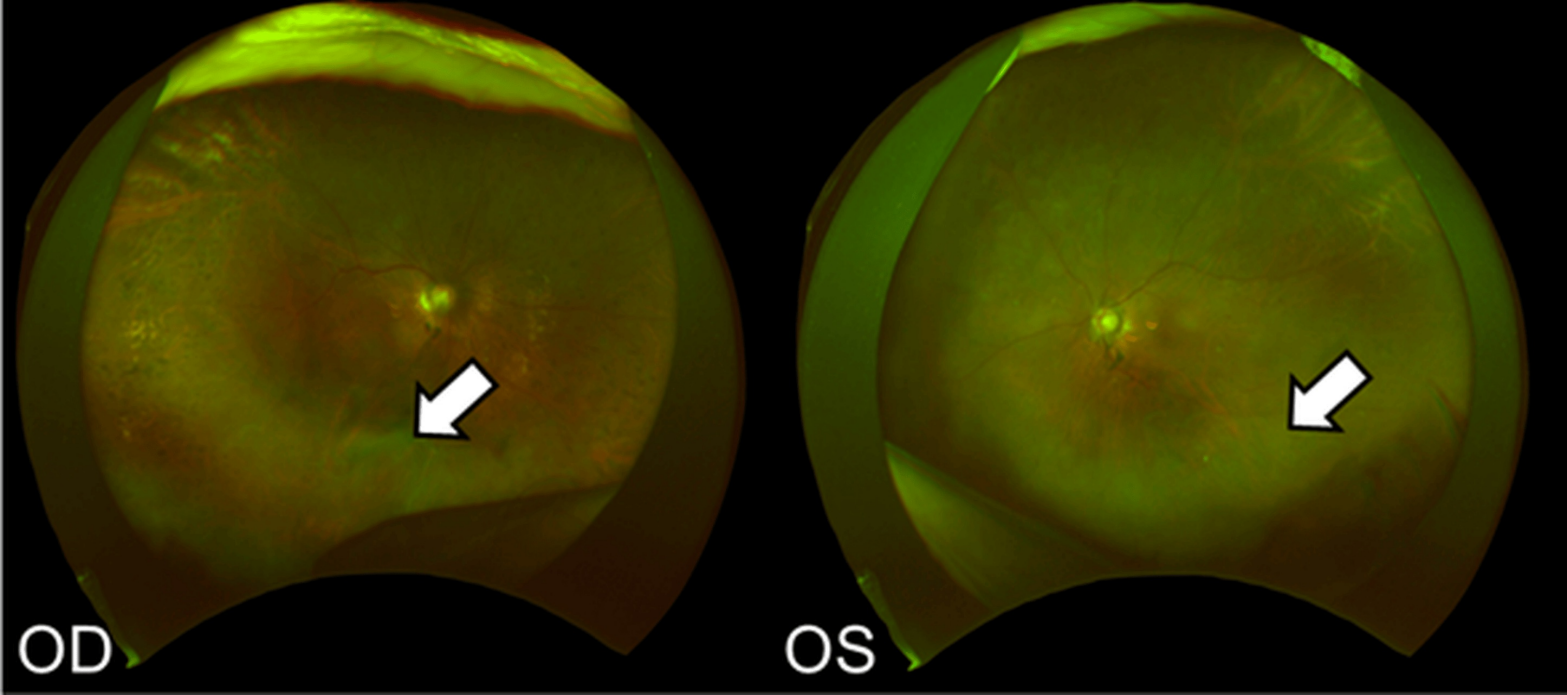
Fundus images one month after the STTA injection Vitreous opacity is improved in both eyes (white arrows). STTA: sub-tenon triamcinolone acetonide; OD: oculus dexter; OS: oculus sinister

Therefore, 20 mg STTA was injected into both eyes. One month later, the vitreous opacity had improved gradually, and BCVA had improved to 20/80 in the right eye and 20/40 in the left eye (Figure 9).

## Discussion

The present study reports three eyes (one eye with AMD and two eyes in the same patient with DME) in two patients that developed IOI following faricimab intravitreal injection, managed with STTA injection. Key findings included keratic precipitates, anterior chamber inflammation, vitreous opacity, and elevated IOP, developing approximately two weeks after the third or fourth faricimab injection (Table 1).

**Table 1:** Clinical characteristics and outcomes of eyes with faricimab-associated IOI nAMD: neovascular age-related macular degeneration; PCV: polypoidal choroidal vasculopathy; DME: diabetic macular edema; IVI: intravitreal injection; IOI: intraocular inflammation; BCVA: best-corrected visual acuity; IOP: intraocular pressure; AC: anterior chamber; SUN: Standardization of Uveitis Nomenclature; STTA: sub-Tenon triamcinolone acetonide; mo: month

Feature	Case 1 (right eye)	Case 2 (right eye)	Case 2 (left eye)
Diagnosis	nAMD (PCV)	DME	
Prior IVI	None	Ranibizumab x3, aflibercept x1	
Faricimab injections (n)	4	3	
Onset after last injection	17 days	14 days	
BCVA at IOI onset (Snellen)	20/80	20/200	20/80
IOP at IOI onset (mmHg)	24	27	25
AC cells (SUN criteria)	2+	2+	2+
Keratic precipitates	Numerous fine, pigmented	Numerous fine	Numerous fine
Vitreous opacity (SUN criteria)	3+	3+	2+
Other findings	Posterior synechiae	-	-
STTA dose	20 mg	20 mg	20 mg
BCVA 1 mo post-STTA (Snellen)	20/25	20/80	20/40
IOP 1 mo post-STTA (mmHg)	17	15	14
Outcome	Inflammation resolved, persistent posterior synechiae	Inflammation improving	Inflammation improving

The differential diagnosis for these findings includes infectious endophthalmitis and herpetic uveitis [11]. Infectious endophthalmitis was considered less likely due to the clinical presentation, which lacked features such as severe pain or hypopyon often associated with bacterial infection and negative bacterial cultures. Aqueous humor PCR testing was performed for all patients and confirmed negative for herpesviruses, supporting a non-infectious etiology. However, as previous reports mention the concomitant use of oral antiherpetic medications in similar cases [11], caution in differentiating IOI from viral uveitis is warranted.

In the TENAYA [7] and LUCERNE [7] nAMD clinical trials, the incidence rates of IOI were 0.3% and 0.9% per person, respectively. In the YOSEMITE [8] and RHINE [8] DME trials, the frequency rates of IOI were 1.0% per person and 0.3% per person, respectively. As noted in the Introduction, these rates from controlled trials may differ from real-world experience, where higher rates have been suggested in some reports [9,10]. In addition, higher incidences of IOI have been reported with a faricimab intravitreal injection than with 2 mg of aflibercept intravitreal injection since the clinical trial stage [7,8], and clinicians should pay attention to IOI when using faricimab.

The exact mechanism underlying faricimab-associated IOI remains unclear. The onset after multiple injections, as observed in our cases (third or fourth injection), might suggest a delayed hypersensitivity reaction or a cumulative immunogenic effect. A non-infectious inflammatory response to the drug molecule or its components is presumed (a possible relevant reference could be added here if available). Unlike the occlusive retinal vasculitis that has been a significant concern with brolucizumab [12-15], the inflammation in our cases primarily involved the anterior chamber and vitreous without definite signs of retinal vasculitis on fundus examination. This suggests that faricimab-associated IOI might have a different clinical spectrum or potentially milder severity compared to brolucizumab-associated events, though severe cases have also been reported [5,6]. However, definitive conclusions about mechanisms or comparative severity cannot be drawn from this small case series.

In this study, early administration of STTA effectively controlled the inflammation and led to visual acuity improvement in all eyes. While posterior synechiae persisted in one eye due to initial severe inflammation, no other serious long-term complications like persistent vision loss or glaucoma were observed during the follow-up period reported. This suggests STTA can be a viable treatment option for managing faricimab-associated IOI, potentially mitigating severe outcomes if administered promptly.

Our report is limited by the small number of cases and relatively short follow-up. This prevents the identification of specific risk factors or definitive characterization of the full spectrum and long-term course of faricimab-associated IOI. Whether factors such as underlying disease (AMD vs. DME), number of prior injections, or patient-specific immune characteristics influence risk requires investigation in larger, prospective studies or registries.

## Conclusions

We report two cases of IOI following multiple intravitreal faricimab injections, presenting with anterior chamber cells, keratic precipitates, and vitreous opacity approximately two weeks post-injection. Herpetic uveitis was excluded by negative PCR testing, supporting a non-infectious, likely drug-related etiology. Early intervention with STTA effectively resolved the inflammation and improved visual acuity in all eyes, although persistent posterior iris synechia remained in one eye, highlighting that complications can occur despite treatment. Compared to reports of IOI associated with brolucizumab, the inflammation observed in these cases appeared milder, without evidence of occlusive vasculitis. Due to the small sample size, further multicenter studies with larger patient numbers are necessary to better characterize the features, incidence, risk factors, and optimal management strategies for faricimab-associated IOI.
